# Supplementary material for: Global hypo-methylation in a proportion of glioblastoma enriched for an astrocytic signature is associated with increased invasion and altered immune landscape
Source: eLife. 2022 Nov 22;11:e77335. doi: 10.7554/eLife.77335 (PMC9681209; doi:10.7554/eLife.77335)
Supplement: Figure 2—source data 1. [file elife-77335-fig2-data1.zip › Figure_2_source_data_1/Figure_2A/homerResults/motif15.similar.html]

motif15

## Information for motif15

T
A
G
C
A
C
T
G
A
C
T
G
A
G
C
T
T
G
C
A
A
G
T
C
A
G
T
C
A
C
G
T
A
C
T
G
G
T
A
C
  
Reverse Opposite:  

A
C
T
G
G
T
A
C
C
G
T
A
C
T
A
G
T
A
C
G
A
C
G
T
T
C
G
A
G
T
A
C
A
G
T
C
A
C
T
G
  

|  |  |
| --- | --- |
| p-value: | 1e-21 |
| log p-value: | -4.864e+01 |
| Information Content per bp: | 1.836 |
| Number of Target Sequences with motif | 19.0 |
| Percentage of Target Sequences with motif | 0.82% |
| Number of Background Sequences with motif | 1.0 |
| Percentage of Background Sequences with motif | 0.03% |
| Average Position of motif in Targets | 88.4 +/- 34.8bp |
| Average Position of motif in Background | 160.0 +/- 0.0bp |
| Strand Bias (log2 ratio + to - strand density) | -0.5 |
| Multiplicity (# of sites on avg that occur together) | 1.00 |
| Motif File: | file (matrix) reverse opposite |

### Similar de novo motifs found

|  |  |  |  |  |  |  |  |
| --- | --- | --- | --- | --- | --- | --- | --- |
| Rank | Match Score | Redundant Motif | P-value | log P-value | % of Targets | % of Background | Motif file |
| 1 | 0.633 | A G T C G A T C G T C A A C G T G C T A T A G C A G T C C G A T A T C G A T G C | 1e-12 | -29.301666 | 3.06% | 1.14% | motif file (matrix) |
| 2 | 0.605 | T A G C G A C T T C G A T G C A T A G C G T A C A T C G T A G C G C T A C T A G T A C G T G C A T A G C G T A C G A T C | 1e-11 | -26.644484 | 0.78% | 0.09% | motif file (matrix) |
